# Use of Smartphone-Based Video Directly Observed Therapy (vDOT) in Tuberculosis Care: Single-Arm, Prospective Feasibility Study

**DOI:** 10.2196/13411

**Published:** 2019-08-27

**Authors:** Samuel B Holzman, Sachin Atre, Tushar Sahasrabudhe, Sunil Ambike, Deepak Jagtap, Yakub Sayyad, Arjun Lal Kakrani, Amita Gupta, Vidya Mave, Maunank Shah

**Affiliations:** 1 Division of Infectious Diseases Johns Hopkins University School of Medicine Baltimore, MD United States; 2 Dr D Y Patil Medical College, Hospital, and Research Center Dr D Y Patil Vidyapeeth Pune India

**Keywords:** Video DOT, mHealth, tuberculosis, medication adherence, telemedicine, India, mobile phone, smartphone

## Abstract

**Background:**

India accounts for nearly one-quarter of the global tuberculosis (TB) burden. Directly observed treatment (DOT) through in-person observation is recommended in India, although implementation has been heterogeneous due largely to resource limitations. Video DOT (vDOT) is a novel, smartphone-based approach that allows for remote treatment monitoring through patient-recorded videos. Prior studies in high-income, low disease burden settings, such as the United States, have shown vDOT to be feasible, although little is known about the role it may play in resource-limited, high-burden settings.

**Objective:**

The goal of the research was to assess the feasibility and acceptability of vDOT for adherence monitoring within a resource-limited, high TB burden setting of India.

**Methods:**

We conducted a prospective, single-arm, pilot implementation of vDOT in Pune, India. Outcome measures included adherence (proportion of prescribed doses observed by video) and verifiable fraction (proportion of prescribed doses observed by video or verbally confirmed with the patient following an incomplete/unverifiable video submission). vDOT acceptability among patients was assessed using a posttreatment survey.

**Results:**

A total of 25 patients enrolled. The median number of weeks on vDOT was 13 (interquartile range [IQR] 11-16). Median adherence was 74% (IQR 62%-84%), and median verifiable fraction was 86% (IQR 74%-98%). More than 90% of patients reported recording and uploading videos without difficulty.

**Conclusions:**

We have demonstrated that vDOT may be a feasible and acceptable approach to TB treatment monitoring in India. Our work expands the evidence base around vDOT by being one of the first efforts to evaluate vDOT within a resource-limited, high TB burden setting. To our knowledge, this is the first reported use of vDOT in India.

## Introduction

Globally, tuberculosis (TB) is the leading cause of infectious disease-related mortality, responsible for 1.6 million deaths annually [1]. The incidence of TB is higher in India than anywhere in the world, with roughly 2.8 million cases reported in 2017, nearly 27% of the global TB burden [1]. To achieve positive treatment outcomes, adherence to TB therapy is critical [2,3]. However, socioeconomic and health system barriers in India are common and negatively impact adherence [4-6]. Failure to complete treatment can lead to relapse and the emergence of multidrug-resistant TB (MDR-TB), resulting in further disease transmission.

The World Health Organization (WHO) encourages the tailored use of multidimensional adherence interventions, including social, material, and psychological support, and emphasizes monitoring through directly observed treatment (DOT) [7]. Compared with self-administered therapy, those managed with DOT have demonstrated an improved rate of treatment completion [7,8]. Completion of therapy is vital not only for the patient but also the community, as public health efforts to mitigate disease spread require treatment success.

Unfortunately, DOT is often burdensome for patients and, paradoxically, can have a negative impact on adherence for some [9]. In India, DOT has historically been largely clinic-based (although there are differences in the public and private sector), wherein patients are required to bear the financial and logistical burden of frequent travel to and from the clinic for treatment monitoring. In doing so, patients risk lost wages due to time away from work. Additionally, providers must record and dispense daily treatments, a process that can be onerous and prohibitive in resource-constrained settings. While DOT is formally recommended under the current TB treatment guidelines set forth by India’s Revised National Tuberculosis Control Program (RNTCP), in practice, DOT implementation (ie, observing and documenting each prescribed dose) in the community is inconsistent, and associated barriers can lead to treatment default [10-15].

More recently, video directly observed therapy (vDOT) has been introduced as a patient-centered alternative to in-person DOT, with pill ingestion monitored remotely via digital video capture. vDOT has been implemented using synchronous technologies [16-19] such as Skype and FaceTime as well as asynchronous technologies [20,21], where recorded videos are uploaded and digitally stored for future review. This latter method allows for video capture to occur at times convenient for the patient and eliminates the need for vDOT to be scheduled around staff availability. Recent work has shown asynchronous vDOT to be feasible, well received by patients and providers, and associated with high rates of treatment adherence [20-27]. Further, two economic evaluations in the United States have suggested vDOT to be cost effective over in-person DOT [20,27]. These encouraging findings have led both the US Centers for Disease Control and Prevention and WHO to suggest vDOT as a viable alternative to in-person DOT [28-30].

While data on vDOT are becoming increasingly robust, vDOT has yet to be rigorously evaluated within low- and middle-income countries of high disease burden such as India. Despite resource constraints, cellular technology has spread rapidly through India. As of 2017, there were a recorded 1.2 billion cellular connections and 291.6 million smartphone users within the country, suggesting that vDOT may have a role in this setting [31,32]. Additionally, recent changes to RNTCP guidelines have prioritized daily therapy (ie, 7 days per week) over three-times-per-week therapy, a change that further questions the feasibility of in-person DOT within a system already stretched thin and underscores the need for alternative approaches to adherence monitoring and support [14,33,34].

To address this critical knowledge gap, we conducted a prospective pilot of vDOT in Pune, India. Specifically, we addressed the feasibility and acceptability of vDOT within this resource-limited setting of high disease burden.

## Methods

### Overview

**Figure 1:**
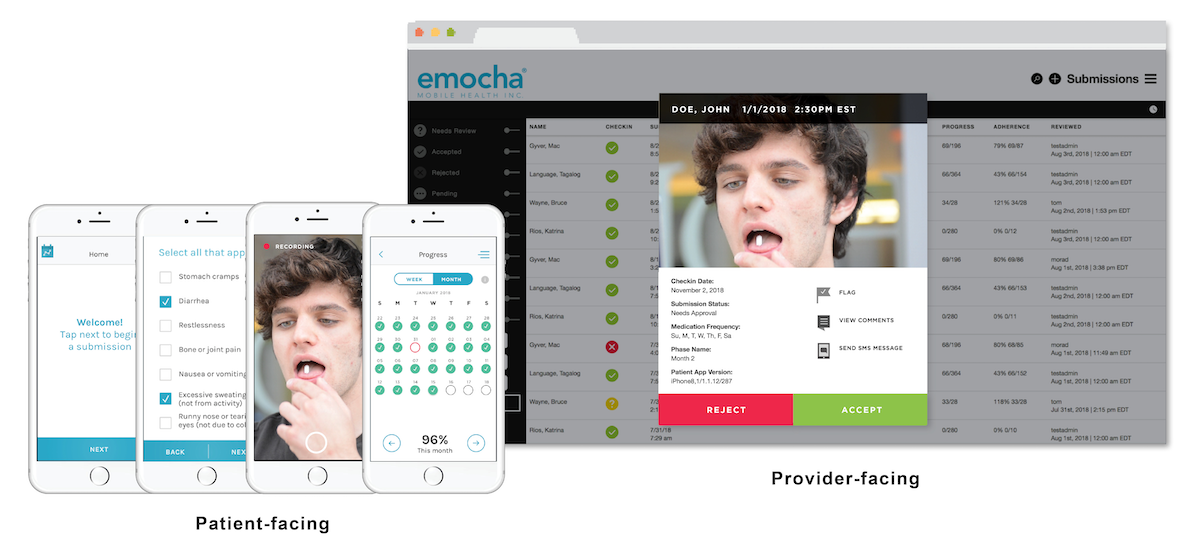
The patient-facing portion of the emocha video directly observed therapy mobile app allows patients to record and transmit treatment videos, report any medication-related side effects, and review treatment progress and track adherence. The provider portion of the platform can be used by medical staff to review treatment videos and accessed from multiple devices.

We conducted a prospective, single-arm, pilot implementation of vDOT in Pune, India. The mobile app emocha vDOT (emocha Mobile Health Inc) was used for treatment monitoring and adherence support (Figure 1). The patient-facing portion of the platform (ie, the mobile app) allows patients to record and transmit treatment videos. The interface also prompts patients to report any medication-related side effects (by checking off relevant symptoms from a prepopulated list). Through a calendar function, patients are able to review treatment progress and track adherence. Use of the software requires a camera-enabled tablet or smartphone device with at least intermittent access to Wi-Fi or cellular data. The app supports both Android and iOS operating systems. The provider portion of the platform can be accessed on a desktop, laptop, tablet, or smartphone (using a mobile browser) and is used by medical staff to review treatment videos. Providers are notified of any patient-reported treatment side effects. Given the system’s asynchronous nature, submitted videos can be reviewed at any time following digital capture and transmission.

**Figure 2:**
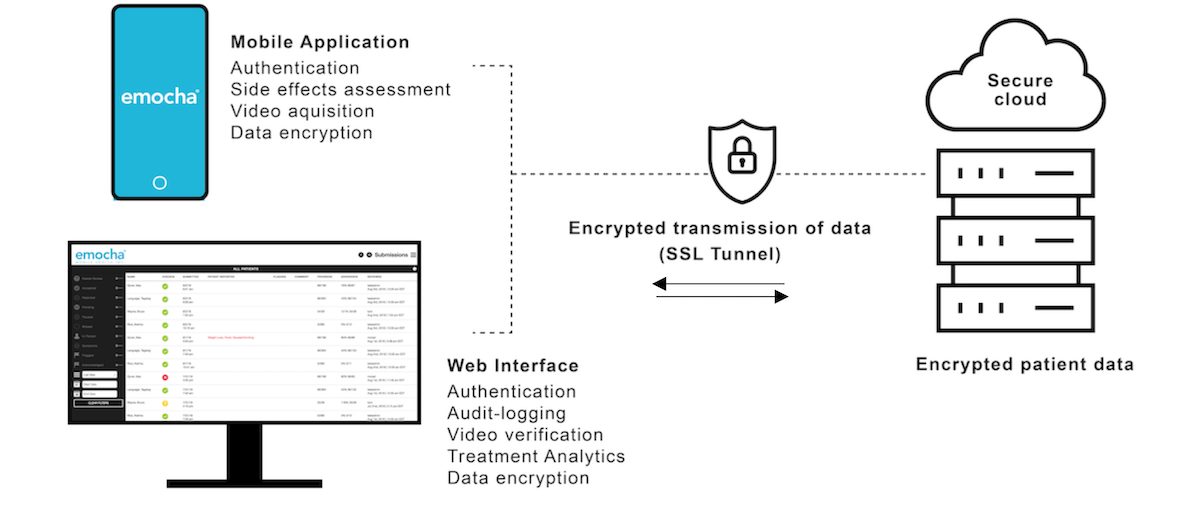
Data flow and security with the emocha video directly observed therapy mobile app.

The emocha app is compliant with US Health Insurance Portability and Accountability Act (HIPAA) regulations and allows for asynchronous vDOT (Figure 2). Video capture occurs via the app. In the event that the device loses internet service or does not have access to internet service during video capture or upload, the videos (or any untransmitted component) remain encrypted on the device; all videos are uploaded automatically to secure servers when connection is restored (Wi-Fi or cellular data). Following transmission, videos are automatically wiped from the smartphone memory. Encrypted patient data, therefore, remain within the device only for the period between video capture and Web upload. Providers are able to access uploaded data via a secure Web interface through which they review submitted videos and track treatment progress.

The study was conducted at the Dr DY Patil Medical College Center and took place between January 2017 and June 2018. Study procedures were approved by the local institutional ethics committee and the institutional review board at Johns Hopkins University in Baltimore, Maryland.

### Participants

Dr DY Patil Medical College Hospital is a private hospital that contains a government (public) TB treatment center (directly observed treatment, short-course, or DOTS center) as a public-private mix initiative. Patients diagnosed with or treated for TB at either Dr DY Patil or local DOTS centers were eligible for the study. Inclusion required age >18 years, signed informed consent, and >2 remaining months of TB therapy. Patients with MDR disease and HIV were excluded. Given this was a pilot study, we enrolled a convenience sample. Some patients were approached at the time of diagnosis, although many were assessed for eligibility midtreatment. Those not participating in the study received treatment and observation as per the local standard of care. Local guidelines recommend DOT for all intensive phase doses and for at least one dose per week during the continuation phase [14], although implementation is heterogenous and largely determined by local resources and patient preference (oral communication, T Sahasrabudhe, MD, November 2018).

Prior to enrollment, patients were required to establish basic smartphone proficiency and demonstrate the ability to successfully navigate the emocha app. A version of emocha translated into Marathi (the primary local language) was available to those with limited English. Patients without access to a smartphone were provided one by the study. Regardless of the device used, each participant was provided Rs 200 (US $3) each month to cover the cost of video submissions and a one-time incentive payment of Rs 100 (US $1.50) to cover travel expenses.

### Study Procedures

A total of 35 patients were selected for this study based on a convenience sampling method. All patients provided written informed consent and were permitted to withdraw from the study at any time. Demographic information including participant medical history and TB diagnosis were collected using a standardized case report form. Data were subsequently entered into a digital database by study staff. During their first study visit, participants were introduced to vDOT by a study staff member who provided each with a unique username and password and conducted a step-by-step tutorial outlining the process for how to create and submit a treatment video. Patients were then observed as they attempted to submit a dummy video independently. Additional training was provided on an as-needed basis.

Prior to formal enrollment, patients underwent a conditional 1-week run-in period, during which they were closely monitored for their continued ability to successfully record and submit videos. Any technical or logistical barriers arising during this period were addressed prior to formal study enrollment, which was only able to occur following successful completion of this trial period. For those enrolled, vDOT continued through treatment completion or until consent was withdrawn. Text message reminders via the emocha app were automatically sent to patients in the absence of expected video submissions. All incomplete or unverifiable videos (eg, medication could not be seen or video did not transmit due to network issue) were followed up with a staff phone call to verbally verify whether the dose was taken.

### Feasibility

Feasibility was assessed by two primary outcomes. The first was treatment adherence, or the proportion of all prescribed treatment doses directly observed by video. As noted above, incomplete or unverifiable videos were followed up with a phone call for verbal verification. As such, a second metric, verifiable fraction, was used to describe the proportion of all prescribed doses that were either directly observed (by video) or verbally confirmed (following incomplete/unverifiable videos). All data analysis was completed in Stata 14 (StataCorp LLC).

### Acceptability

To assess vDOT acceptability among patients, a posttreatment survey was administered comprising a series of categorical and Likert scale questions addressing issues such as mobile phone and internet access, emocha ease of use, convenience, and privacy. To increase our understanding of potential implementation barriers, patients were also informally asked to comment on their experiences and highlight any challenges or concerns they had related to the use of vDOT. Patient responses were noted by study staff at the time of survey administration. Staff were also asked to comment on patient-level barriers observed during the study.

## Results

### Study Participants

**Figure 3:**
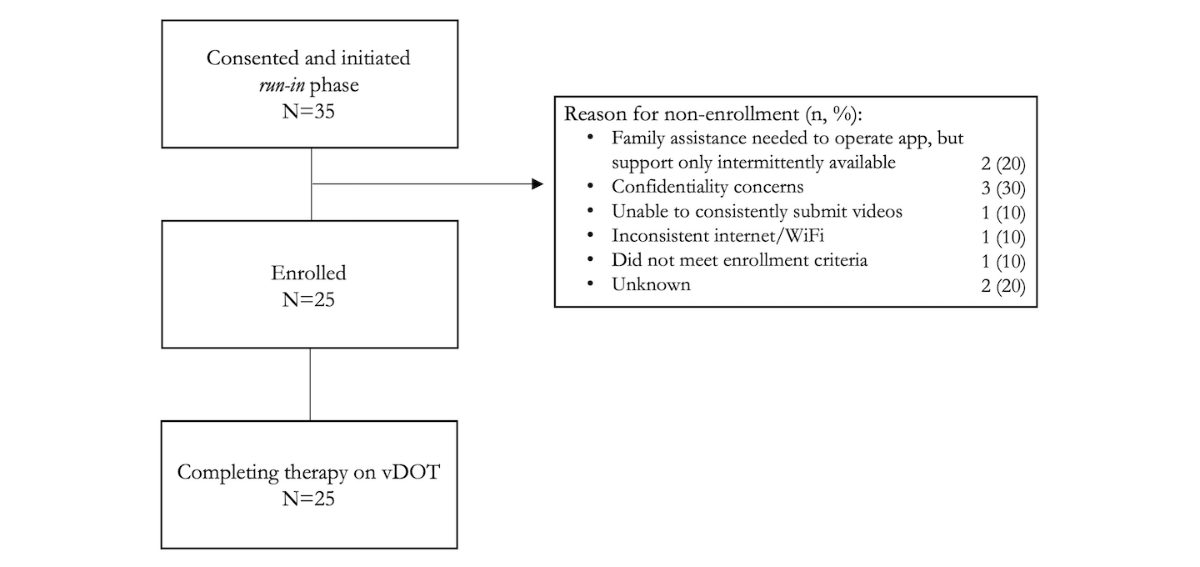
Study flow diagram. vDOT: video directly observed therapy.

Of 35 patients who were consented and initiated the run-in phase (Figure 3), 10 did not complete the run-in and left the study. Reasons for run-in failure were related to technological (eg, inability to effectively use platform or poor cellular/Wi-Fi connectivity) and psychosocial (eg, concerns regarding privacy) barriers. Twenty-five patients were ultimately enrolled and formally initiated on vDOT with emocha. There was no study drop out, and all 25 patients completed therapy on vDOT.

Patient characteristics are described in Table 1. The median age was 27 (interquartile range [IQR] 24-42) years, 40% (10/25) were female, and 72% (18/25) reported their local language as Marathi. Most patients were low income with a monthly income less than Rs 16,000 (US $225). The majority of patients (22/25, 88%) had access to a smartphone and the internet. Three patients (3/25, 12%) required the use of a study phone. Almost three-quarters (18/25, 72%) of patients had pulmonary TB, and the remainder (07/25, 28%) had extrapulmonary disease.

**Table 1:** Patient and disease characteristics (n=25).

Variable			Value
Age, year (median, IQR^a^)			27 (24-42)
Female, n (%)			10 (40)
**Indian state of origin, n (%)**			
	Maharashtra		18 (72)
	Haryana		2 (8)
	Karnataka		1 (4)
	Tamil Nadu		1 (4)
	Other		3 (12)
**Primary language, n (%)**			
	Marathi		18 (72)
	Hindi		6 (24)
	Kannada		1 (4)
Employed, n (%)			10 (40)
**Average monthly income (Rs), n (%)**			
	<2000		6 (24)
	2000-4000		0 (0)
	4000-8000		6 (24)
	8000-16,000		13 (52)
	>16,000		0 (0)
Homeless, n (%)			1 (4)
**Residence, n (%)**			
	Urban		21 (84)
	Rural		4 (16)
Married, n (%)			13 (52)
**Primary mode of transportation, n (%)**			
	Private vehicle		0 (0)
	Bus/train		0 (0)
	Auto-rickshaw		8 (32)
	Other private transportation		17 (68)
**Substance use, n (%)^b^**			
	Alcohol		1 (4)
	Tobacco use		0 (0)
	Illicit drug use		0 (0)
**Medical comorbidities, n (%)^b^**			
	Diabetes		3 (12)
	Hypertension		1 (4)
	Cancer		0 (0)
**Technology, n (%)**			
	Regular access to a smartphone		22 (88)
	Daily access to Wi-Fi or cellular data		22 (88)
	Used personal device for study		22 (88)
**Tuberculosis category, n (%)**			
	**Pulmonary^c^**		
		Smear positive	14 (56)
		Smear negative	4 (16)
	Exclusively extrapulmonary		7 (28)

^a^IQR: interquartile range.

^b^Categories not mutually exclusive, each out of 25 total participants.

^c^Pulmonary disease with or without extrapulmonary involvement.

The majority of patients were initiated on vDOT during the continuation phase (20/25, 80%), with 20% (5/25) beginning during the intensive phase. The median number of weeks on vDOT was 13 (IQR 11-16), with a range of 9 to 23 weeks (Table 2). A total of 80% (20/25) of patients received daily (7 times per week) therapy, while 20% (5/25) received an intermittent (3 times per week) regimen. No in-person DOT was documented either before or after implementation of vDOT. Overall, 60% (15/25) of patients reported at least one treatment-related side effect. The most commonly reported symptoms were nausea/vomiting (8/15), abdominal pain (3/15), and itching (2/15).

**Table 2:** Video directly observed therapy outcomes and data utilization (n=25).

Variable		Value
Adherence^a^ (%), median (IQR^b^)		74 (62-84)
Verifiable fraction^c^ (%), median (IQR)		86 (74-98)
**Dosing frequency, n (%)**		
	3 times per week DOT^d^	5 (20)
	7 times per week DOT	20 (80)
**Treatment phase at enrollment, n (%)**		
	Intensive	5 (20)
	Continuation	20 (80)
Number of weeks on vDOT^e^, median (IQR)		13 (11-16)
Total uploaded videos^f^ (n)		1722
Mean uploads per patient, mean (SD)		91 (53)
**Number of rejected videos per patient**		
	Mean (SD)	1.6 (2.4)
	Range	0-8
Video length (seconds), median (IQR)		44 (31-52)
Video size (MB), median (IQR)		1.5 (1.1-1.7)

^a^Proportion of total prescribed doses completed under video observation. Of note, no in-person directly observed therapy was noted either before or after the implementation of video directly observed therapy.

^b^IQR: interquartile range.

^c^Proportion of total prescribed doses verified by any means, including successful observation by video upload and verbal dose confirmation (by phone or in person) following the submission of an incomplete or poor quality video.

^d^DOT: directly observed therapy.

^e^vDOT: video directly observed therapy.

^f^Total video (accepted + rejected + run-in phase) uploads across all patients over the length of the study.

### Feasibility

Median adherence on vDOT was 74% (IQR 62%-84%, Table 2). After including verbally verified doses (following unverifiable or incomplete videos), the median verifiable fraction was 86% (IQR 74%-98%). An average of 91 (SD 53) videos were submitted per patient. The average number of rejected videos per patient was 1.6 (SD 2.4), with 56% (14/25) having no rejected videos at all. The most common reasons for video rejection were poor quality of video and medication not fully seen. The median video length was 44 (IQR 31-52) seconds and associated with a median file size of 1.5 (IQR 1.1-1.7) MB.

### Acceptability

A total of 22 posttreatment surveys were completed; 3 patients declined participation. Study outcomes for those declining involvement were similar to those of the general study population; each patient completed >14 weeks on vDOT with an adherence >70%.

A total of 91% (20/22) of surveyed patients described emocha as easy to use (Table 3). All patients (22/22, 100%) reported being able to record videos without difficulty, 95% (21/22) were able to upload without difficulty, and 91% (20/22) found text message reminders helpful. Further, all found they were able to communicate concerns and medication side effects effectively through the emocha platform. The majority felt vDOT would be more convenient (20/22, 91%) and preferred (20/22, 91%) over in-person DOT (Table 4). While 82% (18/22) felt vDOT would preserve patient privacy over in-person DOT, 18% (4/22) disagreed and felt in-person DOT would be more private.

**Table 3:** Responses from patient agreeability survey (n=22).

Survey statements (rated on a 5-point Likert scale)	Agree^a^ n (%)	Disagree^b^ n (%)
emocha was easy to use	20 (91)	2 (9)
I was able to record videos without difficulty	22 (100)	0 (0)
I was able to upload videos without difficulty	21 (95)	1 (5)
emocha text message reminders were helpful	20 (91)	2 (9)
I was able to communicate concerns and side effects using emocha effectively	22 (100)	0 (0)

^a^Agree/strongly agree were grouped.

^b^Neutral/disagree/strongly disagree were grouped.

**Table 4:** Responses from patient preference survey (n=22).

Survey statements (categorical)		Value, n (%)
**Videos were most often uploaded using**		
	Wi-Fi at the clinic	0 (0)
	Wi-Fi at home or other location	0 (0)
	Cellular data (3G/4G)	22 (100)
**Which better preserves patient privacy?^a^**		
	vDOT^b^	18 (82)
	In-person DOT^c^	4 (18)
	No preference	0 (0)
**Which is more convenient?^a^**		
	vDOT	20 (91)
	In-person DOT	2 (9)
	No preference	0 (0)
**Preference for therapeutic monitoring^a^**		
	vDOT	20 (91)
	In-person DOT	2 (9)
	No preference	0 (0)

^a^In-person directly observed therapy (DOT), either prior to enrollment or while on video directly observed therapy (vDOT), was inconsistently performed and/or documented based on chart reviews. Answers referring to in-person DOT are therefore based on patient perceptions of what in-person DOT would be like.

^b^vDOT: video directly observed therapy.

^c^DOT: directly observed therapy.

Study coordinator notes were reviewed and summarized in Table 5. Broadly, these notes revealed patient-level barriers impacting the successful implementation and use of vDOT. Included were psychosocial factors, such as the privacy concerns and stigma, and mental health barriers. Despite survey data suggesting that most were able to record and upload videos without issue, poor connectivity and cellphone-related challenges (eg, subscriber identity module [SIM] card malfunction) were noted in a few cases.

**Table 5:** Patient-level barriers to successful video directly observed therapy use as identified by study staff.

Barrier to vDOT^a^ use		Representative patient quotes and/or problem details
**Psychosocial**		
	Stigma	“Recently one of my close relatives expired. As you know, we need to be at home to complete all the rituals up to 15 days after death. All the relatives are there, around all the time, and it became difficult to go out as well. So I could not take videos. Otherwise they would have started asking. Due to that, sometimes I missed my medicines.”
	Hospital admission	One patient suffered from severe alcohol dependence. The patient was successful on vDOT for a period but later admitted for detoxification. The patient’s phone was confiscated at the time of admission, leaving him unable to upload videos during his hospital stay.
	Stress	“My 1-year-old son fell from the bed and his hand got fractured. He was unwell, so we were under stress. I took tablets but during that time, I did not record videos.”
**Technology-related**		
	Connectivity	“I went to my village for 8 days for some work. As we do not have range and connectivity to the internet, I could not send videos.”
	vDOT-related challenges	“The registration process is a bit complicated and time-consuming. Can it be simplified?”
		“The [vDOT] app got hanged in my mobile. I did not know how to reinstall it. So I could not send videos.”
		“When [recording a] video, if I get a call, the application used to suddenly shut down. So the video [would get lost].”
	SIM^b^ card	“I did not submit Know Your Customer documents required for SIM verification. Hence my SIM card was deactivated for some time...I was not able to send videos.”

^a^vDOT: video directly observed therapy.

^b^SIM: subscriber identity module.

## Discussion

### Principal Findings

Our pilot study suggests that vDOT may be a feasible option for verification of medication adherence for TB patients in India. Among enrolled participants who completed a short run-in period to assess technological literacy, we found that a median 74% of all prescribed doses were observed. Further, when including doses verbally confirmed (following incomplete video submissions), the proportion of verified doses (verifiable fraction) increased to 86% (based on 1722 submitted and reviewed videos), exceeding the adherence goal of >80% set forth by current treatment guidelines [28]. This degree of adherence is comparable to that described using vDOT in other settings, such as the United States, and advances current evidence supporting vDOT, as prior work has largely focused on implementation within resource-rich settings [16,20,27,35]. To the best of our knowledge, this is the first reported use of vDOT in India.

Our demonstration of vDOT feasibility within the Indian context is both timely and critical given the recent RNTCP guideline changes emphasizing the need for daily over intermittent (3 times per week) therapy [14,33,34]. While a DOTS strategy, based on the principle of direct treatment observation, has been in place in India for over two decades, in practice, DOT implementation has been inconsistent.

In Pune, our experience has been that patients are often provided medication weekly or biweekly, with adherence monitoring largely based on self-report. At best, clinic services, including in-person DOT, are generally available 6 days per week, permitting a maximum of only 85% of prescribed (daily) doses to be observed. In contrast, by decoupling video capture from provider review, asynchronous vDOT potentially allows for all (100%) doses to be observed and obviates the need to coordinate DOT around staff availability.

To successfully and sustainably implement DOT in India, alternatives to in-person DOT are clearly needed. vDOT has the potential to be this alternative and to fill the needed gap. Our study is among the first in a resource-limited setting to demonstrate that daily therapy can be confirmed through the use of innovative mobile technologies. vDOT saves health care worker time and obviates the need for in-person visits to observe treatment [22]. For settings where home visits are employed solely for DOT, vDOT may reduce costs and save time even further [18,20,27,36,37]. vDOT may also have other previously unrecognized benefits related to infection control. Provisions for personal protective equipment (ie, masks for health care workers) or environmental controls (isolation rooms) are limited in India; vDOT offers a mechanism to closely monitor patients while reducing potential transmission opportunities. Additionally, we observed that patients derived benefit from avoiding frequent clinic visits, for which associated travel leads to lost time and, often, wages. Most importantly, vDOT provides solid evidence of treatment adherence. Our study also highlights a need for patient training (eg, run-in period with onboarding to the technology), counseling, and follow up in cases of missed doses to assure successful treatment completion.

Of note, India has already endorsed another electronic form of treatment monitoring, 99DOTS: when a patient removes a pill from a blister pack, a number is revealed that completes a toll-free phone number printed on the pack, which the patient then calls to report having taken daily medication [12,33]. While 99DOTS may be a feasible means for basic adherence monitoring [38], vDOT has the distinct advantage of providing video confirmation of pill ingestion. It is also important to consider that the use of vDOT allows for adherence support in addition to adherence tracking. The platform used in this study captures side effects and TB symptoms, and videos can also be used to notify providers of treatment concerns, such as rashes, which can be preliminarily evaluated from afar through submitted videos. Moreover, the current platform allows automated messaging reminders, which patients reported to be a benefit. Newer versions of the software offer secure chat functionality (with health care providers) and case management tools that may further support treatment adherence. India recently rolled out a direct benefits transfer scheme that encourages treatment adherence through the use of financial incentives (Rs 500 per month while on therapy) [39,40]. 99DOTS is currently being used as a mechanism to monitor treatment adherence, but it is limited. For the reasons noted above, a more reliable tamper-proof means of adherence monitoring would be beneficial.

### Limitations and Strengths

While our work supports further evaluation of vDOT within India, we acknowledge several study limitations. First, our sample size was small and, while we have shown vDOT to be feasible in one location, its acceptability and feasibility in other parts of India remain unknown. Second, we were unable to compare adherence on vDOT to that under the existing standard of care, which at our site was primarily self-administration (thus precluding documentation of prestudy adherence). Our findings, however, suggest that vDOT implementation could substantially improve adherence documentation compared with current practice. Through broader implementation, vDOT has the potential to enable enhanced accountability among TB clinics with regard to treatment adherence. Improvements in documentation would also increase the availability of high-quality data on TB treatment completion for public health reporting practices. Whether vDOT is associated with improved patient outcomes compared with standard of care is still unknown and was not assessed within the scope of this pilot study.

We also acknowledge a significant attrition over the course of our run-in period. One-third of those who consented did not ultimately participate in the study. Drop out during this period was largely driven by technological barriers related to infrastructure (eg, inconsistent cellular coverage) or inability/unease with smartphone operation. Further, despite the fact that we used a HIPAA-compliant app (emocha) with stringent security controls, several participants withdrew consent over privacy concerns. Some patients noted a fear that their treatment videos might end up publicly viewable on the internet. While cellphone technology has spread rapidly across India, cellular coverage remains incomplete and not all have become immediately facile with the technology. With time, these barriers may diminish. A strength of our study was the use of a run-in period, which was advantageous in that it allowed for rapid identification of those with sufficient mobile phone literacy to be candidates for vDOT. In our study, all those who completed the run-in period and enrolled in the study successfully finished therapy on vDOT.

### Conclusions

Despite its promise, there remain questions regarding vDOT that must be addressed. Larger controlled and comparative trials will be needed to better evaluate the effectiveness of vDOT against the current standard of care or alternative technologies in resource-limited, high disease burden settings. Future studies addressing cost and cost effectiveness are also needed. Last, in other settings such as the United States, vDOT has successfully been coupled with individualized case management to allow real-time intervention after missed doses; the role of this approach in India is unknown [20]. Overall, our work has shown that despite socioeconomic and structural barriers, vDOT may be a feasible approach for treatment monitoring in India.

## Abbreviations

DOT: directly observed treatment
DOTS: directly observed treatment, short-course
HIPAA: Health Insurance Portability and Accountability Act
IQR: interquartile range
MDR-TB: multidrug-resistant tuberculosis
RNTCP: Revised National Tuberculosis Control Program
SIM: subscriber identity module
TB: tuberculosis
vDOT: video directly observed therapy
WHO: World Health Organization
